# Eicosanoid-Activated PPARα Inhibits NFκB-Dependent Bacterial Clearance During Post-Influenza Superinfection

**DOI:** 10.3389/fcimb.2022.881462

**Published:** 2022-07-04

**Authors:** Ronald Lucarelli, Norma Gorrochotegui-Escalante, Jessica Taddeo, Bettina Buttaro, Joris Beld, Vincent Tam

**Affiliations:** ^1^ Center for Microbiology and Immunology, Lewis Katz School of Medicine, Temple University, Philadelphia, PA, United States; ^2^ Sol Sherry Thrombosis Research Center, Lewis Katz School of Medicine, Temple University, Philadelphia, PA, United States; ^3^ Department of Microbiology and Immunology, Center for Advanced Microbial Processing, Institute for Molecular Medicine and Infectious Disease, Drexel University College of Medicine, Philadelphia, PA, United States

**Keywords:** influenza, *Staphylococcus aureus*, eicosanoid, Cytochrome P450, lipidomic, innate immunity, Superinfection

## Abstract

Secondary bacterial infection (superinfection) post influenza is a serious clinical complication often leading to pneumonia and death. Eicosanoids are bioactive lipid mediators that play critical roles in the induction and resolution of inflammation. CYP450 lipid metabolites are anti-inflammatory lipid mediators that are produced at an excessive level during superinfection potentiating the vulnerability to secondary bacterial infection. Using Nanostring nCounter technology, we have defined the targeted transcriptional response where CYP450 metabolites dampen the Toll-like receptor signaling in macrophages. CYP450 metabolites are endogenous ligands for the nuclear receptor and transcription factor, PPARα. Activation of PPARα hinders NFκB p65 activities by altering its phosphorylation and nuclear translocation during TLR stimulation. Additionally, activation of PPARα inhibited anti-bacterial activities and enhanced macrophage polarization to an anti-inflammatory subtype (M2b). Lastly, *Ppara* ^–/–^ mice, which are partially protected in superinfection compared to C57BL/6 mice, have increased lipidomic responses and decreased M2-like macrophages during superinfection.

## Introduction

Influenza virus, an enveloped, negative-sense, single-stranded RNA virus, is an important human pathogen. Influenza infection predisposes the host to secondary bacterial infection. This superinfection is a clinically significant problem and a major cause of mortality and morbidity. Superinfection with *Staphylococcus aureus* following influenza leads to severe disease with approximately 41% mortality (Hageman et al., 2006). *S. aureus* is a Gram-positive bacterium estimated to be carried by 20% of the population (Kluytmans et al., 1997). The emergence and prevalence of MRSA (methicillin-resistant *S. aureus*) and VRSA (vancomycin-resistant *S. aureus*) have significantly increased the threat posed by these bacteria (Kobayashi et al., 2012).

Secondary bacterial infection occurs as the immune system is resolving the influenza-induced inflammation. While the induction of inflammation has been the subject of active research, the mechanisms underlying the resolution of inflammation have remained elusive. Induction of inflammatory response ensures successful pathogen clearance. Resolution of inflammation, on the other hand, returns the immune system to homeostasis thus avoiding excessive tissue damage (Serhan et al., 2015). Eicosanoids are bioactive lipids that play critical roles in both the induction and resolution of inflammation (Dennis and Norris, 2015). During microbial insult or cellular injuries, arachidonic acids and other related polyunsaturated fatty acids, like eicosapentaenoic acids (EPA) and docosahexaenoic acids (DHA), are metabolized *via* three major metabolic pathways, Cyclooxygenase (COX), Lipoxygenase (COX), and CYP450, to produce hundreds of lipid species with diverse physiological activities.

We have previously characterized the lipidomic landscape during the induction and resolution of inflammation in mice and human patients who were infected with influenza (Tam et al., 2013). Eicosanoids are bioactive lipids acting as signaling molecules that play a major role in both the induction and resolution of inflammation (Tam, 2013; Dennis and Norris, 2015). Eicosanoid metabolism pathways have provided highly successful targets for pharmaceutical interventions: non-steroidal anti-inflammatory drugs (NSAIDs) inhibit the cyclooxygenase pathway (COX) (Simmons et al., 2004), while asthma and COPD drugs inhibit the lipoxygenase pathway (LOX) (Scow et al., 2007; Bruno et al., 2018). Subsequently, we have applied systems biology approaches to define the transcriptional and lipidomic responses in a mouse model of influenza/*S. aureus* superinfection (Tam et al., 2020). We identified an anti-inflammatory eicosanoid response (CYP450 lipid mediators) that was highly induced during superinfection. CYP450 lipid mediators activate the nuclear receptor and transcription factor PPARα which can affect the regulatory networks of other transcription factors *via* protein-protein interactions. During influenza single infection, a moderate induction of CYP450 during the resolution phase which may allow for an appropriate anti-inflammatory response to promote the return to homeostasis. In contrast, during *S. aureus* single infection, a minimal level of CYP450 metabolites was produced. Therefore, transcription factors mediating pro-inflammatory signaling ensure successful pathogen clearance. However, excessive induction of CYP450 during superinfection leads to the suppression of innate immune response thus inhibiting efficient bacterial clearance. As *S. aureus* persists, the lipidomic response amplifies the infiltration of inflammatory cells, which eventually causes excessive tissue damage and increased mortality. Interestingly, excessive CYP450 lipid mediators have been observed in COVID patients with severe disease (Schwarz et al., 2021). The pathological production of these lipid mediators may dysregulate the physiological process of resolving inflammation and exacerbate morbidity and mortality during microbial infections.

Macrophages play an essential role in both immunity lipid homeostasis through their scavenger ability to phagocytose microbes or lipids in their resident tissues (Rigamonti et al., 2008). When macrophages are exposed to specific lipids, activated receptors can change the pathological states associated with the local environment (Rigamonti et al., 2008). Some of the common nuclear receptors (NR) include glucocorticoid receptors (GR) or estrogen receptors (ER), but macrophages also have a retinoid-x receptor (RXR) called the peroxisome proliferator activated receptor (PPAR) (Rigamonti et al., 2008). PPAR has three isoforms- PPARα, PPARδ/β, and PPARγ, which are ligand dependent transcription factors that bind to peroxisome proliferator response elements (PPRE) that are in enhancer sites of specific genes (Berger and Moller, 2002). Among immune cells, PPARα is specifically present and highly expressed in peripheral mononuclear immune cells like macrophages (Rakhshandehroo et al., 2010).

PPARα has been shown to play a critical role during microbial infections. In a mouse *Mycobacterium abscessus* infection model, *Ppara* ^–/–^ knockout mice show notably higher bacterial loads and increased cytokine expression of pro-inflammatory genes, including *Il6, Il1b*, and *Cxcl10* (Kim et al., 2020). Furthermore, macrophage polarization profiles can be influenced by PPAR activation. Infection of macrophages with the obligate intracellular parasite, *Trypanosoma cruzi*, increased classically activated (M1) markers (e.g. NOS2) and increased proinflammatory cytokine signaling (Penas et al., 2015). Activation of PPARγ with 15dPGJ2 or PPARα with WY14643 showed increased Arginase-1 (M2 marker) and decreased pro-inflammatory cytokine expression (Penas et al., 2015). These studies demonstrate that activation of PPAR promote an anti-inflammatory phenotype.

While we demonstrated the role of CYP450 during superinfection *in vivo*, the mechanisms by which the lipid mediators affect the molecular signaling and cellular function on inflammation and bacterial clearance are not well understood. In this study we investigated the impact of CYP450-PPARα axis on the inflammatory signaling in macrophages since they are the dominant cell types in the broncho-alveolar lavage during superinfection. Using Nanostring nCounter Technology, we determined that the CYP450 lipid metabolites dampened the TLR inflammatory transcriptional responses in macrophages. We demonstrated that the activation of PPARα inhibits NFκB, hinders antibacterial activities and modulates macrophage polarization. Lastly, using liquid chromatography-Mass spectrometry (LC-MS), we determined the lipidomic profiles in wild type and *Ppara* ^–/–^ mice during superinfection. The increased eicosanoid metabolism in *Ppara* ^–/–^ mice may contribute to increased survival in during superinfection.

## Results

### Activated PPARα Inhibits NFκB Activity and Pro-Inflammatory Gene Expression

**Figure 1:**
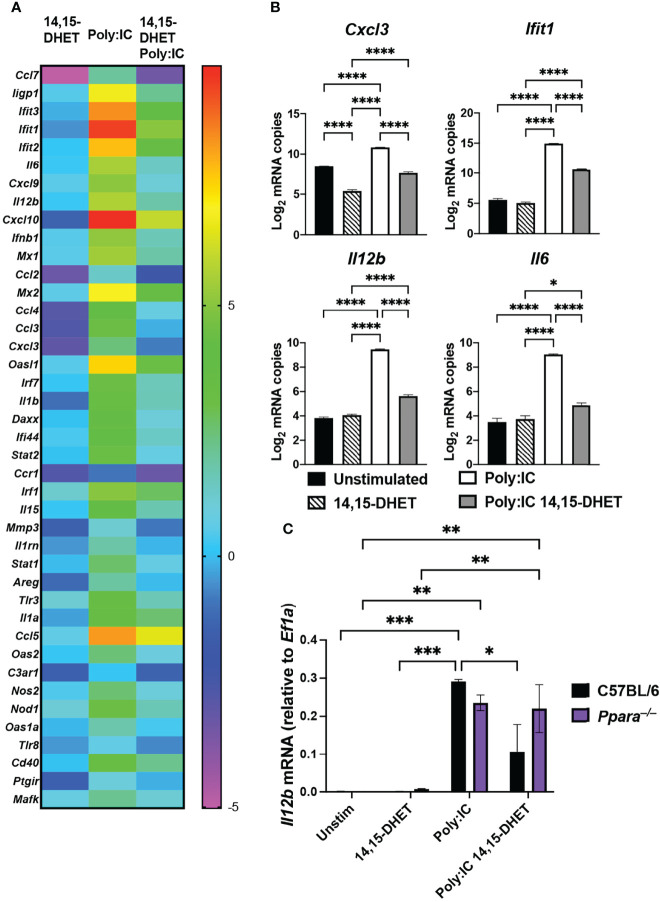
PPARα activation dampens pro-inflammatory gene expression in macrophages. **(A)** Heat map depicts fold changes of transcript levels in C57BL/6 macrophages stimulated with 14,15 DHET, poly:IC (TLR3 agonist), or 14,15 DHET with poly:IC normalized to unstimulated for 3h. RNA was extracted and analyzed using Nanostring nCounter Technology (Inflammation panel of 254 mouse genes including 15 internal reference genes). n=3 per group. Data was analyzed using nSolver software. Genes displayed have P value < 0.01, FDR<0.05. **(B)** Bar graphs depict the log2 expression fold change of *Cxcl3*, *Il6*, and *Il12b*, (cytokines/chemokines) and *Ifit1* (interferon regulated gene) relative to unstimulated cells for the indicated conditions. **(C)** Bar graph depicts transcript levels (mean +/- SEM) of *Il12b* levels as measured by RT-PCR from Hoxb8 macrophages (C57BL/6, black or *Ppara* ^–/–^, purple) stimulated with 14,15-DHET, poly:IC, or 14,15 DHET with poly:IC normalized to *Ef1a*. Two way ANOVA with multiple comparisons were performed to determine statistical significance (*P ≤ 0.05; **P ≤ 0.01; ***P ≤ 0.001; ****P ≤ 0.0001).

We have previously determined that increased production of CYP450 metabolites during superinfection have reduced pro-inflammatory genes induction in cells isolated from Broncho-alveolar lavage or whole lung lysates (Tam et al., 2020). Since a majority of the BAL cell population consisted of inflammatory monocytes, macrophages and neutrophils, we determined to dissect the molecular mechanism by which CYP450 lipid metabolites hinder the proinflammatory response in macrophages. We determined the targeted transcriptional response of bone marrow-derived macrophages after polyinosinic:polycytidylic acid (poly:IC) stimulation by Nanostring nCounter Technology. We determined that after the addition of 14, 15 DHET (dihydroxy-eicosatrienoic acid, a CYP450 lipid metabolite), genes related to inflammatory response are induced to significant lower levels than poly:IC stimulated macrophages (**Figure 1A**; **Supplementary Figure 1A**). The genes with blunted response to the TLR3 ligand (**Supplementary Figure 1B**, cluster 1) include cytokines and chemokines (*Ccl5, Ccl7*, *Cxcl3, Cxcl10*, *Il6*, *Il12b*), type I interferon regulated genes (*Mx1*, *Mx2*, *Ifit1*, *Ifit2*, *Ifit3*, *Oasl1*) (**Figures 1A, B**). Moreover, the effects of 14,15 DHET is *Ppara*-dependent in which the induction of *Il12b* from poly:IC stimulation was not affected by the CYP450 lipid metabolite in *Ppara* ^–/–^ cells (**Figure 1C**). From these data, we concluded that activation of CYP450 lipid metabolite directly hinders the TLR signaling of macrophages. Next, we performed promoter enrichment analysis (HOMER v4.11) to determine candidate transcription factor mediating the suppression of inflammatory response. Using hierarchical clustering to identify genes that are induced during *S. aureus* infection and significantly suppressed during superinfection, we determined that the top enriched motif to be that of the transcription factor, NFκB p65 (P value 1e-6, q-value 0.0001, **Supplementary Figure 1C**). We utilized a luciferase reporter (Wilson et al., 2013) transduced into Hoxb8 macrophages (macrophages differentiated from Hoxb8-driven conditionally immortalized myeloid progenitors) (Wang et al., 2006) generated from C57BL/6 and *Ppara* ^–/–^ mice to assess the NFκB activity during TLR stimulation with or without PPARα activation. While TLR stimulation significantly increased NFκB activities, activation of PPARα hampered the activities in wild type C57BL/6, compared to *Ppara* ^–/–^ (**Supplementary Figures 2A, B**). Unexpectedly, *Ppara* ^–/–^ did not induce significant NFκB activity upon poly:IC or LPS (TLR4 ligand) stimulations (**Supplementary Figures 2A, B**). Since PPARα plays additional roles including fatty acid metabolism, the lack of PPARα in *Ppara* ^–/–^ macrophages may alter the threshold for immune response. These data suggest that activation of PPARα with either CYP450 lipid metabolite or synthetic ligand WY14643, the chemical agonist with comparable activity to 14,15 DHET (Fang et al., 2006), suppresses NFκB activities and hence the inflammatory response in macrophages. We have used WY14643 in lieu of CYP450 lipid metabolites for its specificity and feasibility, and we have selected concentration by activation efficacy and ensuring cytotoxicity does not occur (**Supplementary Figure 2C**).

### Activated PPARα Alters NFκB Localization and Phosphorylation

**Figure 2:**
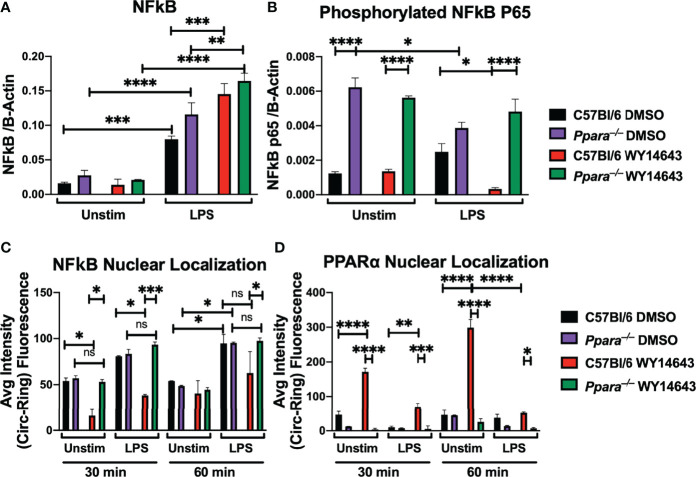
PPARα activation hinders NFκB p65 phosphorylation and nuclear translocation during TLR stimulation. Bar graphs depict protein quantifications (mean+/- SEM) from immunoblotting of wild type C57BL/6 or *Ppara* ^–/–^ Hoxb8 macrophages stimulated with LPS (TLR4 agonist) for 30 minutes with or without WY14643 (PPARα agonist) against NFκB **(A)** and phosphorylated NFκB p65 **(B)**. Bar graphs depict nuclear localization (mean+/- SEM) of NFκB **(C)** and PPARα **(D)** in wild type C57BL/6 or *Ppara* ^–/–^ Hoxb8 macrophages stimulated with LPS (TLR4 agonist) for 30 or 60 minutes with or without WY14643 (PPARα agonist). Images were analyzed by HCS software for nuclear localization. Two way ANOVA with multiple comparisons were performed to determine statistical significance (*P ≤ 0.05; **P ≤ 0.01; ***P ≤ 0.001; ****P ≤ 0.0001; ns not significant), n=3 and are representative of 3 experiments.

To further determine the mechanism by which activation of PPARα affects NFκB, we used automated digital microscopy and immunoblotting to determine the localization and abundance of NFκB p65. NFκB is a master transcription factor involved in inflammation and cell death (Huang et al., 2010). Upon stimulation *via* TLR by microbial infection or specific TLR ligands, NFκB p65 is phosphorylated, which promotes nuclear translocation (Oeckinghaus and Ghosh, 2009). To assess how activation of PPARα suppresses NFκB activities, we determined the phosphorylation status of NFκB during TLR stimulation with or without PPARα synthetic agonist. While the abundance of NFκB was similar between conditions, abundance of phosphorylated NFκB p65 was decreased during TLR stimulation with WY14643 in wildtype macrophages (**Figures 2A, B**, **Supplementary Figure 3A**). Moreover, the phosphorylation status of macrophages generated from *Ppara* ^–/–^ animals did not differ with the PPARα agonist. Using automated digital microscopy, we performed immunofluorescent microscopy to determine the nuclear translocation of both NFκB and PPARα. Upon stimulation with TLR ligand (LPS) with or without WY14643, macrophages were immediately fixed and permeated, followed by stained with DAPI, anti-NFκB p65 (CY-5), and anti-PPARα (AlexaFluor 488) antibodies. Nuclear localization of p65 was significantly decreased when WY14643 was administered with LPS compared to TLR ligands alone (**Figure 2C**; **Supplementary Figures 4**, **5**). While wildtype macrophages stimulated with PPARα agonist present with distinct nuclear localization of PPARα, knockout macrophages showed a faint fluorescence outside the nucleus (**Figure 2D**, **Supplementary Figures 4**, **5**). The residual signal in the *Ppara* ^–/–^ macrophages can be explained by the non-specific binding of anti-PPARα antibodies in both wildtype and *Ppara*–/– cells (**Supplementary Figure 3B**). The protein analysis and localization studies suggest that the activation of PPARα decreased the phosphorylation and nuclear localization of NFκB.

### PPARα Activation Dampens Phagocytosis and Bacterial Clearance

**Figure 3:**
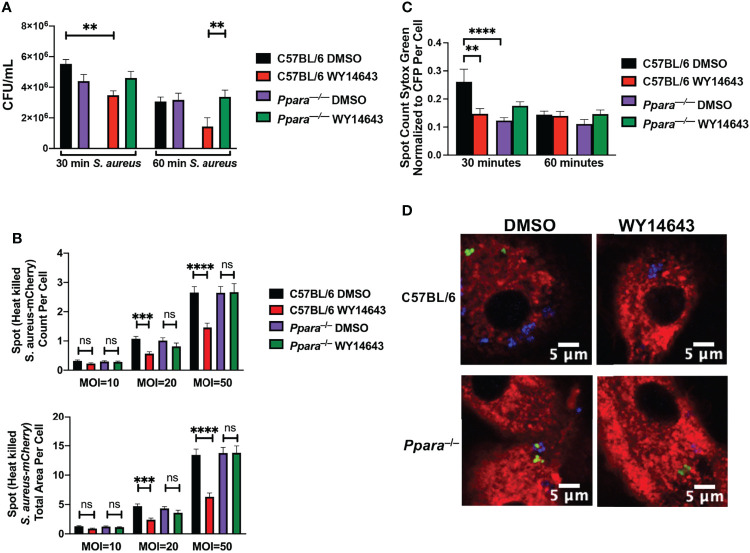
Phagocytosis and bacterial killing are dampened during PPARα induction. **(A)** Bar graph depicts Colony Forming Units (mean+/- SEM) in BMDM macrophages stimulated with DMSO or WY14643 and infected with *S. aureus* at an MOI 20 for 30 or 60 minutes. **(B)** Bar graphs depict spot count (averaged count of bacterial cells in population of cells of each microscopy image, mean+/- SEM) and total area of Heat killed CFP-expressing *S. aureus* phagocytosed by macrophages at 30 minutes. **(C)** Bar graph depicts spot count (averaged count of bacterial cells in population of cells of each microscopy image, mean+/-SEM) of Sytox Green (cell death marker) positive normalized to CFP-expressing *S.aureus* within macrophages at 30 or 60 minutes post infection with or without WY14643. Two way ANOVA with multiple comparisons were performed to determine statistical significance (**P ≤ 0.01; ***P ≤ 0.001; ****P ≤ 0.0001; ns not significant). **(D)** Representative digital images of macrophages (cell membrane stained with CellMask Orange, red), live *S. aureus* (CFP, blue) and dead *S. aureus* (Sytox Green, green). n=3 per group and are representative of 3 experiments.

Since we observed persistent bacterial colonization in the lungs of influenza/*S. aureus* super-infected animals, we hypothesized that CYP450-PPARα axis hinders the phagocytic or bactericidal activities of macrophages. To understand the physiological role of PPARα activation in phagocytosis and bacterial clearance, we infected C57BL/6 and *Ppara^-/-^* macrophages derived from murine bone marrow or Hoxb8 macrophages of each genotype (data not shown) with *S. aureus* for 30 and 60 minutes, washed the macrophages to isolate the intracellular populations, and determined the colony forming units. Activation of PPARα by WY14643 resulted in lowered bacterial burden in macrophages (**Figure 3A**). While the CFU assay can assess the intracellular bacterial loads at the given time points, the dynamic host-pathogen interactions cannot be discerned. To address whether the difference in CFU was due to differences in phagocytosis or bacterial killing, we used digital and confocal microscopy to determine uptake and bactericidal activities. We introduced heat-killed CFP-expressing *S. aureus* and used automated digital microscopy and High Content Screening analysis software to determine phagocytosis by macrophages with or without PPARα activation by WY14643. While wild type C57BL/6 and *Ppara* ^–/–^ macrophages were able to phagocytose similar level of heat killed bacteria at 30 minutes and 1 hour, activation of PPARα by WY14643 inhibited phagocytosis in wild type but not *Ppara* ^–/–^ macrophages (**Figure 3B**). To determine bacterial killing activities of macrophages, we infected macrophages with CFP-expressing live *S. aureus* and stained the bacteria with Sytox green (**Figures 3C, D**). Live bacteria will not retain the fluorescence from Sytox Green while killed bacteria, due to the loss of its membrane integrity, will be stained. Using CellMask orange and DAPI to counterstain the plasma membrane and nucleus, respectively, we conducted both confocal microscopy and automated fluorescent microscopy. We observed significant bacterial killing by wild type macrophages at 30 minutes post-infection while activation of PPARα by WY14643 significantly decreased the ability of macrophages to kill *S. aureus* (**Figures 3C, D**). This killing inhibition by WY14643 was also not observed in *Ppara* ^–/–^ macrophages. These data suggest that activation of PPARα in macrophages hinders the phagocytic and bactericidal activities. Furthermore, the phagocytosis and clearance capabilities are not affected in *Ppara* ^–/–^ macrophages by PPARα agonist.

### PPARα Influences Macrophage Polarization and Lipidomic Responses During Superinfection

**Figure 4:**
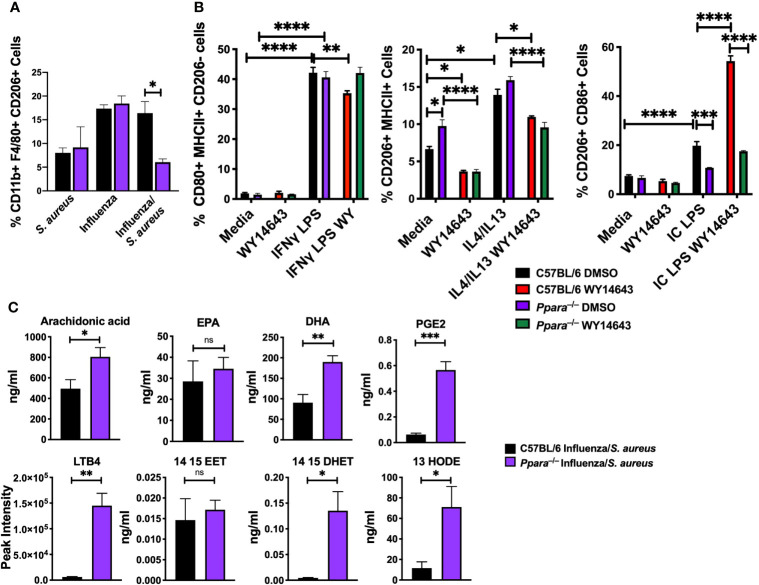
PPARα knockout is protective against influenza/*S. aureus* superinfection. **(A)** Bar graph depicts percentages of CD11b+ F4/80+ CD206+ M2 macrophages (mean+/-SEM) from *S. aureus*, influenza, or influenza/*S. aureus* infected C57Bl/6 (black) or *Ppara* ^–/–^ (purple) mice on day 8 post-infection. N=3-5, representative of 3 independent experiments. **(B)** Bar graphs depict percentages of C57BL/6 (black (DMSO) and red (WY14643)) and *Ppara^-/-^* (purple (DMSO) and green (WY14643)) BMDM macrophages polarized to M1, M2a, and M2b phenotypes, with or without the presence of PPARα agonist, WY14643. Two way ANOVA with multiple comparisons were performed to determine statistical significance (*P ≤ 0.05; **P ≤ 0.01; ***P ≤ 0.001; ****P ≤ 0.0001). n=3 per group and are representative of 3 experiments. **(C)** Lipidomic mass spectrometry measurements from broncho-alveolar lavage of immune-associated lipids (arachidonic acid, EPA, DHA, PGE2, 14,15-EET, 14,15-DHET, LTB4, 13 HODE, and 15d PGJ2). Student’s T tests were performed to determine statistical significance (*P ≤ 0.05; **P ≤ 0.01; ***P ≤ 0.001; ns not significant). n=4 per group.

Macrophages play an important role in the induction and resolution of inflammation. The activities of macrophages depend on the microenvironment and autocrine/paracrine signaling. Macrophages can polarize into M1 (classical), M2a (alternatively activated), M2b (anti-inflammatory), and M2c (wound healing) subsets (Sica and Mantovani, 2012; Arora et al., 2018; Wang et al., 2018). PPAR has been shown to modulate M1 M2 macrophage polarization. While PPARγ has been shown to affect macrophage polarization (Nelson et al., 2018; Yao et al., 2018), the role of PPARα remains elusive (Penas et al., 2015). While wild type animals all succumb to superinfection, *Ppara* ^–/–^ animals were partially protected (Tam et al., 2020). Cellularity studies of collected broncho-alveolar lavage (BAL) post-infection (on day 8) determined that there were significantly fewer M2 macrophages in *Ppara^–/–^* mice during superinfection compared to wild type animals (**Figure 4A**; **Supplementary Figure 6**). Additionally, as there were comparable populations of inflammatory monocytes, DC, T cells, B cells and NK cells between wild type and *Ppara^–/–^* mice, there was an increase of neutrophils within the *Ppara^–/–^* mice **(** **Supplementary Figures 6**, **7** **).** To understand how PPARα affects macrophage polarization, we skew bone marrow-derived macrophages to M1 with LPS and IFNγ for 24 to 48 h, M2a with IL4/IL13, M2b with Immune Complex (Ova and anti-Ova) and LPS, and M2c with IL10/TGFβ. When we polarized macrophages by subtype stimuli and activated PPARα with WY14643, there was a significant decrease in M1 macrophages that was dependent on *Ppara* (**Figure 4B**). This effect does not show switching from M1 to M2 macrophages exclusively. M2a polarization was also slightly decreased with PPARα activation. Interestingly, M2b macrophages were significantly enhanced by WY14643 that was dependent on *Ppara*. We observed no difference with M2c polarization (data not shown). Using *Il12b* (M1), *Arg-1* (M2a), and *IL10* (M2b) as polarization markers, we have demonstrated that PPARα activation directly reduces the M1 and M2a populations while increasing the M2b population (**Supplementary Figure 8**). Moreover, as macrophage polarization results in a spectrum of phenotypes rather than strict qualitative changes, we determined that the mean fluorescent intensity of CD206, a mannose receptor expressed on M2 macrophages (Tsuchiya et al., 2019), was significantly increased in C57BL/6 macrophages with the addition of WY14643 but not during M2a polarization, or in *Ppara* ^–/–^ macrophages (**Supplementary Figure 9**). These data suggests that activation of PPARα specifically increased the M2b populations both qualitatively and quantitatively. The eicosanoid metabolic response is highly dynamic due to transcellular metabolism (a collaboration of different cell types participating in eicosanoid production) and metabolic shunt (inhibition or down-regulation of an enzyme within one pathway may “shunt” the substrate through another pathway) (Buczynski et al., 2009; Norris and Dennis, 2012). We conducted lipidomic profiling (using LC/MS/MS) in C57BL/6 and *Ppara* ^–/–^ mice during superinfection to determine how the genetic perturbation affects the lipidomic responses (**Figure 4C**). Comparative lipidomic profiling between wildtype and the *Ppara* ^–/–^ knockout mice illustrate an altered population of lipid mediators are produced influencing the inflammatory response during superinfection. Major precursors (including arachidonic acids and DHA), cyclooxygenase -derived PGE2, lipoxygenase-derived LTB4, CYP450 metabolite (14,15 DHET) and linoleic acid-derived metabolite (13 HODE) were significantly increased in *Ppara* ^–/–^ mice, compared to C57BL/6. These data suggest that PPARα activation influences the macrophage polarization *in vitro* and *in vivo*. In addition, the resulting altered macrophage function and eicosanoid metabolism may contribute to the differences in mortality and morbidity in superinfection, where *Ppara* ^–/–^ mice are partially protected.

## Discussion

Influenza is an important human pathogen causing serious clinical complications. Prevalence and mortality vary greatly depending on the circulating seasonal or pandemic virus strain. Using a non-biased global systems biology approach and focusing on eicosanoids, we have identified a subset of lipid mediators that are produced during the resolution of inflammation (Tam et al., 2020). These eicosanoids are natural ligands for the nuclear receptors/transcription factors PPAR, which play critical roles in regulating macrophage polarization, inflammation, and lipid metabolism (Ng et al., 2007). To understand the mechanisms by which the CYP450-PPARα axis exacerbates mortality and morbidity during superinfection, we investigated how activation of PPARα affects the inflammatory signaling in macrophages. Using targeted transcriptional profiling with Nanostring nCounter Technology, we determined that CYP450 metabolites dampens the TLR signaling pathway *via* PPARα activation. We assessed the transcriptional activities of NFκB with lentiviral luciferase reporters. Activation of PPARα with CYP450 metabolite or synthetic ligand decreased NFκB activities. Moreover, activation of PPARα inhibits NFκB p65 phosphorylation and nuclear translocation.

PPAR has been shown to modulate the transcriptional networks by multiple mechanisms (Ricote and Glass, 2007; Pawlak et al., 2015). PPAR can directly interact with other transcription factors (i.e. p65) and prevent binding to NFκB response element or activating transcription. Activated PPAR can also induce IκBα which inhibits NFκB in the cytoplasm. Additionally, activated PPAR can regulate kinase activity, compete for coactivator complex or even inhibit co-repressor clearance.

The precise mechanism by which activated PPARα inhibits NFκB will be elucidated in future studies. We used both poly:IC and LPS to highlight that regardless of signaling through TLR3-TRIF or TLR4-TRIF and MyD88, the effects of CYP450 lipid-PPARα axis acts further downstream of the signaling cascade which may be significant due to the difference in PAMPs detected in multi-pathogenic interactions with the host immunity in superinfection.

High levels of CYP450 lipid mediators were detected in influenza superinfection as well as in serum from patients with severe COVID disease (Schwarz et al., 2021). Besides hindering the anti-bacterial activities, CYP450 lipid mediators decimated the anti-viral response, including numerous Type I interferon response and regulated genes (*Ifnb1*, *Mx1*, *Mx2*, *Oasl1*, *Ifit1*, *Ifit2*, *Ifit3*) (**Figures 1A, B**). The blunted anti-viral response may allow the unrestrained replications of viral pathogens which lead to an eventual cytokine storm (Ragab et al., 2020). Concordantly, immunosuppressed adaptive and innate immune cells and early-stage immune suppression have been observed from COVID patients (Remy et al., 2020; Tian et al., 2020). In contrast, the production of CYP450 lipid mediators occur during the resolution phase of influenza (7-10 days post infection), the dampening of antiviral response would allow for the return to homeostasis while adaptive immunity continues to eradicate the virus.

While we observed a persistence of *S. aureus* in the lungs of influenza-infected mice (Tam et al., 2020), using an *in vitro* system, we determined that the anti-bacterial function of macrophages was greatly hindered due to PPARα activation (**Figure 3**). By utilizing CFU assays and digital microscopy, we were able to quantify a diminished phagocytic capability during PPAR activation. Digital microscopy using fluorescent markers to distinguish killed bacteria further showed that bacterial killing functions were also hampered during infection. Macrophages greatly affect the mediation of inflammation from the onset to resolution of infection. Interestingly, activation of PPARα is protective against *Mycobacterium tuberculosis* and *Pseudomonas aeruginosa* infections (Kim et al., 2017; Gugliandolo et al., 2019). While *Ppara* ^–/–^ mice have increased cytokine expression of pro-inflammatory genes during bacterial infection, as similarly observed during influenza/*S. aureus* superinfection, *Ppara* ^–/–^ mice have increased bacterial loads during *M. tuberculosis* (MTB) and *P. aeruginosa* infections. While MTB and *P. aeruginosa* are restricted and controlled by autophagy, *S. aureus* induces and exploits autophagy for its survival and growth (Schnaith et al., 2007; Huang and Brumell, 2014). Since PPARα plays a role in modulating autophagy (Jiao et al., 2014; Lee et al., 2014), the difference in phenotypes in *Ppara* ^–/–^ mice may be explained by the pathogenic mechanisms of the pathogens.

**Figure 5:**
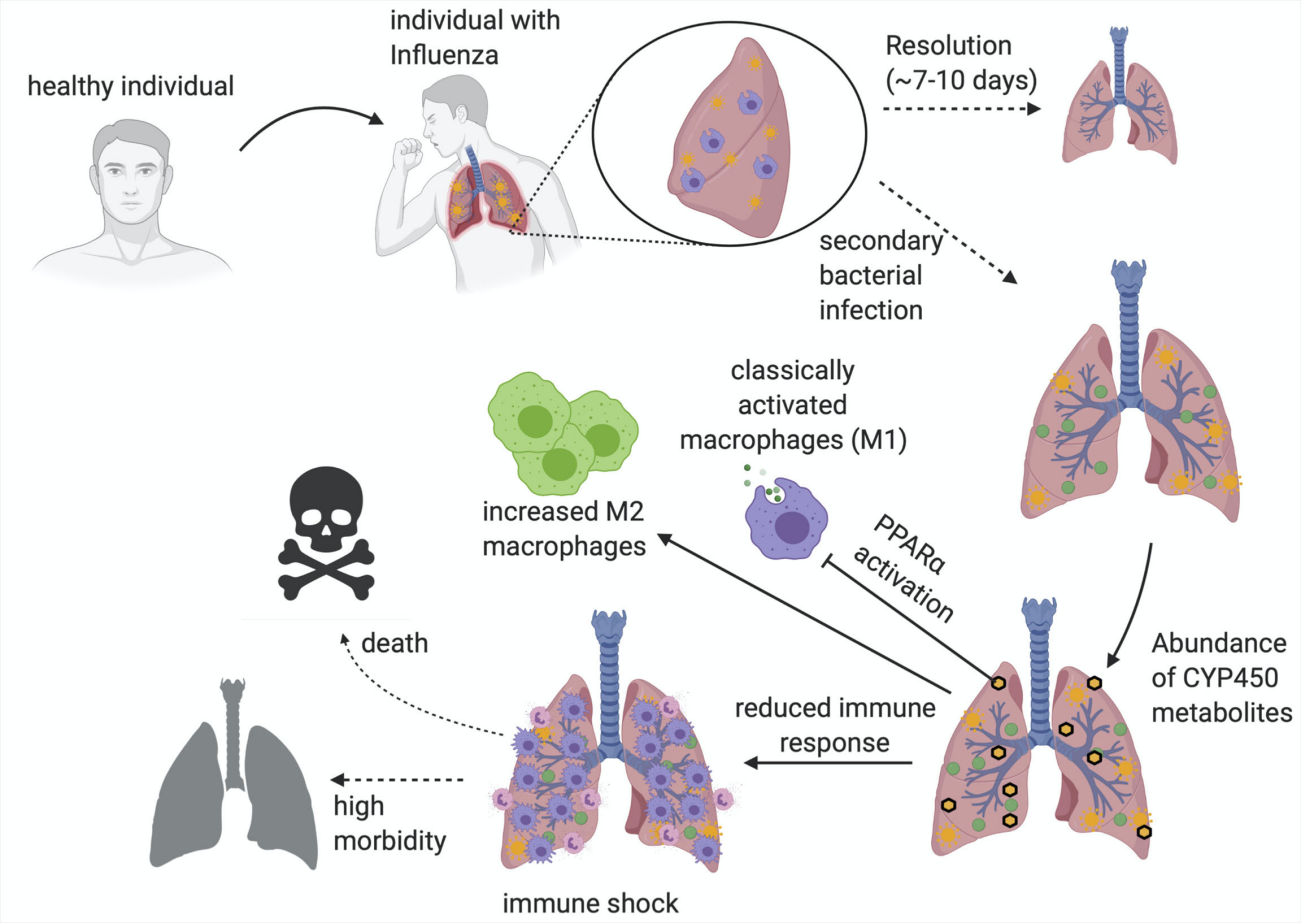
An overview of superinfection. An illustrated image depicting the progression of post-influenza secondary bacterial superinfections. The major hallmarks of infection revolve around the dampening of immune response, the subsequent immune shock, and the outcome of increased morbidity and mortality. Illustration created with BioRender.com.

Since the elucidation of a specific transcription factor (GATA-3) promoting CD4 Th2 subset differentiation, mirroring M1 and M2 macrophage polarization have been defined to play diverse roles in health and disease (Zheng and Flavell, 1997; Mantovani et al., 2004). During superinfection, we have determined that there was a significant increase in alternatively activated M2 macrophages during the resolution of superinfection (**Figure 4A**). Using an *in vitro* polarization to the M2b subtype, enhancement of M2b (induced by immune complex/TLR ligand) polarization by PPARα agonist is intriguing because generation of anti-influenza IgG antibodies begins around day 7 post primary infection (Doherty et al., 2006). Increased polarization to M2b may play a critical role for effective dampening of the immune response *via* IL10 to prevent tissue damage. During influenza infection, the production of anti-influenza antibodies occurs during the resolution phase of inflammation which is also when the host is vulnerable to superinfection (**Figure 5**). While influenza infection has been shown to promote alveolar macrophages to M1/M2b phenotype (Zhao et al., 2014), M2 macrophages have been shown to play diverse roles during superinfection: STAT2 deficiency increased M1, M2, and M1/M2 macrophages which promoted bacterial clearance (Gopal et al., 2018); SHP2 deficiency increased M2 macrophages and hindered antibacterial activities (Ouyang et al., 2020).

The effects of CYP450-PPARα axis on macrophage polarization, anti-bacterial activities, and abilities to recruit other immune cells (particularly neutrophils), may be exploited by the bacterial pathogens during superinfection (**Supplementary Figure 4**). When PPARα is activated during superinfection, macrophages have reduced immune response involving cytokines, such as Cxcl3 (**Figure 1**), and eicosanoids, such as LTB4 (**Figure 4C**). These signaling molecules play critical roles in recruitment of neutrophils (Zhang et al., 2001; Afonso et al., 2012; Wu et al., 2012; Lämmermann et al., 2013). While neutrophils are recruited in knockout mice during superinfection, the dampening of Cxcl3 and LTB4 correlates with decreased infiltration into the lungs of wildtype mice. This is problematic for the host immune system that has decreased macrophage functions in both immune response and function (namely phagocytosis and bacterial killing). Neutrophils and macrophages both play an essential role during influenza pneumonia infections as the main immune cell infiltrates during infection (Rudd et al., 2019). When uncontrolled recruitment and activation of neutrophils occur during influenza infection, the exacerbated outcomes are altered due to acute lung injury caused by excessive infiltration and generation of NETs (Rudd et al., 2019). However, the regulatory network to prevent exacerbated immune response may benefit the opportunistic secondary bacterial infection. Besides LTB4, other eicosanoids, such as PGE2, which has both pro- and anti-inflammatory activities (Sander et al., 2017), and 13-HODE (anti-inflammatory lipid mediator) (Vangaveti et al., 2010), were significantly increased during superinfection in *Ppara* ^–/–^ mice. Moreover, while 14, 15 EET (precursor to 14,15 DHET) was produced at similar levels, 14,15 DHET was produced at a significantly higher level in *Ppara^–/–^* mice, compared to wild type. The increased lipidomic response of *Ppara^–/–^* mice may be due to the lack of anti-inflammatory signals *via* CYP450-PPARα axis. The increased lipid mediators produced in *Ppara* ^–/–^ mice suggests that the CYP450-PPARα axis limits the eicosanoid metabolism. This negative feedback loop may be signaled *via* the transcriptional changes, immune cell activation status, or indirectly *via* altered fatty acid metabolism which generates the precursors for eicosanoids. The precise mechanism by which PPARα affects eicosanoid metabolism will be investigated in future studies.

The shift in anti-bacterial activities, macrophage polarization, and lipidomic responses may explain the difference in mortality and morbidity during superinfection, where *Ppara* ^–/–^ mice are partially protected. When individuals are infected with influenza, resolution of inflammation occurs approximately around 7-10 days (**Figure 5**). The activation of PPARα promotes a systemic dampening of inflammatory response, propagating a cascade that affects macrophage function and immune cell recruitment to assure successful resolution. The resultant dampening of the immune response during resolution is sometimes exploited by a secondary bacterial infection post-influenza. Due to this anti-inflammatory immune environment, the macrophages are not activated to properly respond to the infection, nor recruit the proper immune cell response (i.e., neutrophils). The failure to control the bacterial infection ultimately leads to an immune shock that will severely impact the pathology of the lung, possibly causing death (**Figure 5**).

PPARα has been demonstrated in other infection and disease models to exert an anti-inflammatory or repair activity. For example, activation of PPARα can reduce the inflammatory effects of LPS-induced acute lung injury by ameliorating vascular leakage and release of cytokine and eicosanoids into the alveolar space (Schaefer et al., 2008). Activation of PPARα can also restore the mitochondrial structure and promote gut epithelial repair during SIV infection in a nonhuman primate (Crakes et al., 2019). Moreover, PPARα is a master regulator for lipid metabolism (Bougarne et al., 2018). The activation of PPARα may modulate the metabolism which alters the immune function and activity of the cells. Surprisingly, we observed a decreased NFκB activity during TLR stimulation and an absence of increased bacterial killing in *Ppara* ^–/–^ macrophages. The lack of PPARα in the *Ppara* ^–/–^ macrophages may alter the threshold for immune response. The mechanisms by which CYP-PPARα affect the immunometabolism will be elucidated in future studies. Interestingly, while fenofibrates (PPARα agonist) are prescribed for abnormal blood lipid levels, infection and pneumonia are cited as possible adverse side effects (FDA, 2008; FDA, 2010) and fenofibrate has been associated with higher risk of mortality in MTB infections (Liu et al., 2020). In contrast, in a mouse influenza model, administration of oseltamivir (antiviral) and fibrates (PPARα agonist) prolong survival time during lethal H7N9 infection (Xu et al., 2015). Similarly, administration of gemfibrozil (fibrates) also increased survival during H2N2 infection (Budd et al., 2007). From these data, fibrates had been proposed as an inexpensive treatment against severe influenza infections or influenza pandemic (Fedson, 2009). Our data may explain the seemingly contradictory concepts. PPARα agonists may be useful for successful resolution during influenza infection (**Figure 5**). However, during superinfection, the pathological production of DHET excessively activates PPARα, compromising the immune system’s ability to control secondary bacterial infections (**Figure 3**). If fibrates therapy drastically increase the activation of PPAR, patients may be at risk for superinfection. However, the use of fibrates is confounded by the fact that it also inhibits CYP2C enzymes (Gong et al., 2016) which may prevent dangerous levels of DHET. Furthermore, data is not available to assess PPAR activation in the lungs of patients under fibrates therapy. And the level of PPAR activation may not be increased at a pathological level for bacteria to cause opportunistic infections.

The complexity of the superinfection stems from a triad of interacting players: the host immune response, influenza virus, and the bacterium. Successful pathogen clearance and resolution of inflammation ensure physiological return to homeostasis. In contrast, dysregulated functions resulting from aberrant PPAR activation will hinder pathogen control and eventually amplify inflammation. Combining systems approaches, targeted molecular methods, and high throughput cell imaging, we have determined how the CYP450-PPARα axis potentiates the increase of morbidity and mortality during superinfection. Understanding the molecular mechanism by which resolution of inflammation affects our immune response will yield therapeutic targets for sever microbial infections and inflammation-mediated diseases.

## Materials and Methods

### Mouse Influenza and *Staphylococcus aureus* Infection

C57BL/6J and *Ppara* ^–/–^ (Stock No: 008154) mice were obtained from Jackson Laboratory (Bar Harbor, ME). Experiments were approved by the Temple University IACUC. Infection groups were 10 animals each, 5 male and 5 female randomly selected among 8-12 week age group from our holding colony. Mice were either infected with influenza, *S. aureus*, or both viral and bacterial infection (superinfection). Animals were anesthetized with a ketamine/xylazine mixture and infected intranasally with 100 PFU of influenza virus strain PR8 in 30µl sterile PBS. Mock-infected animals were inoculated with 30µl sterile PBS. Animals were weighed and monitored daily. *S. aureus* (Newman) at 1x10^7^ CFU/20µl was inoculated *via* non-invasive intratracheal inoculation (DuPage et al., 2009). Eight (8) days post-infection, mice were euthanized and both lungs and broncho-alveolar lavage using sterile PBS was collected for further analysis.

### Animal Husbandry

Animals are kept under the veterinary care of the Temple University Laboratory and Animal Resources (ULAR) department. Mice are assessed for health and safety each day and are provided fresh food and water by animal husbandry staff. The facility undergoes a 12-hour daylight, 12-hour nighttime cycle. The Temple University Institutional Animal Care and Use Committee (IACUC) has approved our experimental and care approaches for bone marrow harvesting (5002) and our superinfection model (5000).

### Hoxb8 Macrophages Stimulation

Macrophages were stimulated with poly:IC (6µg/ml, LMW, *In vivo*gen) or LPS (10µg/ml, *Salmonella minnesota* R595, List Biological Laboratories). Cells were pretreated with vehicle (DMSO) or WY14643 (100µM, Sigma Aldrich), or 14, 15-DiHETrE (10µM, Cayman Chemical). WY14643 is a specific PPARα chemical agonist and is a feasible substitute used instead of the purified lipids.

### RNA Extraction and qRT-PCR

After stimulation of macrophages, RNA was extracted by TRIzol (Invitrogen) and Direct-zol 96 RNA Preps (Zymo Research). cDNA was synthesized using random hexamer and TaqMan Reverse Transcription Reagents (Applied Biosystems). TaqMan Fast Advance Master mix and TaqMan Primer/Probe sets were used for qRT-PCR in ABI StepOne System (Applied Biosystems).

### Hoxb8 Macrophages Transduced With Luciferase Reporter

NFκB luciferase reporter, pHAGE NFκB-TA-LUC-UBC-GFP-W, was a gift from Darrell Kotton (Addgene plasmid # 49343; http://n2t.net/addgene:49343; RRID: Addgene_49343). NFκB reporter construct, Δ8.9, and pCMV-VSVG were transfected into 293T (ATCC) using TransIT^®^-Lenti Transfection Reagent (Mirus Bio LLC). Supernatant containing lentivirus was harvested 48 hours post transfection and incubated at 4°C overnight after diluting with 40% PEG8000, 2M NaCl pH7.2. Lentivirus was concentrated by ultracentrifugation at 16,000rpm for 30’. Pellet was resuspended transduced into Hoxb8 progenitors using polybrene. Transduced cells were FACS sorted with Influx (BD Biosciences).

### Luciferase Assay

After stimulation of Hoxb8 macrophages, cell lysates were analyzed using Luciferase 1000 Assay System according to manufacturer (Promega). Plates were analyzed using BMG Labtech Omega Plate reader.

### Automated Digital Microscopy

Cells were seeded into 384 or 96 well #1.5 glass bottom plate (Nunc or Cellvis). Images were captured using EVOS 2 FL (Invitrogen) and analyzed using HCS Studio Cell Analysis Software (ThermoFisher).

### NFκB and PPARα Nuclear Translocation

C57Bl/6 and *Ppara^-/-^* macrophages were first stimulated with WY14643 for one hour to activate PPARa, followed by TLR stimulation *via* LPS for 30 or 60 minutes to induce the NFκB cascade. Cells were fixed with 2% PFA, then permeated with 0.1% Triton-X for 10 minutes for intracellular staining. Cells were blocked with blocking buffer (PBST, 1%BSA, 22mg/mL glycine) for 30 minutes, then stained with primary antibodies for NFκB p65 (Santa Cruz Biotechnology, SC-8008, 1:100) and PPARα (Novus Biologicals, NR1C1 (pSer12), 1:100). Secondary staining goat anti-mouse IgG Cyanine 5 (ThermoFisher, A10524, 1:1000) and goat anti-rabbit Alexa Fluor™ 488 (ThermoFisher, A11054, 1:1000) were used. DAPI (Cayman Chemical, 14285, 300nM) was used for nuclear staining. Cells were imaged using EVOS 2 FL (Invitrogen) and analyzed using HCS Cell Studio Analysis Software (ThermoFisher).

### Protein Analysis

Protein lysates were separated by electrophoresis (Tris-Glycine SDS PAGE). Transferred PVDF membranes (LI-COR) were stained with anti-NFκB p65 (F-6, Santa Cruz), anti-PPARα (MA1-822, ThermoFisher), or anti-actin (C4, Santa Cruz). Secondary antibodies IRDye 800CW and IRDye 680RD were used and protein bands were detected using LI-COR Odyssey and analyzed using ImageStudio (LI-COR).

### CFU Assays

BMDM or Hoxb8 macrophages were plated stimulated with vehicle DMSO (Sigma Aldrich, D2650) or WY14643 (Sigma-Aldrich C7081) for 1 hour, then biological triplicates were infected with *S. aureus* Newman-mCherry for 30, 60, 90, or 120min. 24 well plates were immediately centrifuged at 200 x g for 5 minutes. Macrophages were washed 3x with PBS, then lysed with 0.1% Triton-X and plated in serial dilutions on TSA plates. Colony formation was manually counted and analyzed using Graphpad Prism (San Diego, CA).

### Phagocytosis- Microscopy

Macrophages were stimulated with vehicle DMSO (Sigma Aldrich, D2650) or WY14643 for 60 minutes, then biological triplicates were infected at an MOI of 10, 20, or 50 with heat-killed *Staphylococcus aureus* Newman-CFP for 30, 60, 90, or 120 minutes. 24 well plates were immediately centrifuged at 200 x g for 5 minutes. Macrophages were fixed at end of experimental trial and stained with DAPI (Cayman Chemical, 14285, 300nM), then imaged using the EVOS 2 FL (Invitrogen) and analyzed with HCS Cell Studio Analysis Software (ThermoFisher) to detect fluorescent bacteria within stained macrophages.

### Anti-Bacterial Killing- Microscopy

Macrophages were stimulated with vehicle DMSO (Sigma Aldrich, D2650) or WY14643 for 60 minutes, then biological triplicates were infected at an MOI of 20 with live *Staphylococcus aureus* Newman-mCherry for 30, 60, 90, or 120 minutes. 24 well plates were immediately centrifuged at 200 x g for 5 minutes. Macrophages were stained with CellMask™ Orange (#C10045) just prior to infection, and Sytox™ Green (S7020, 0.4mM) to detect bacterial killing. Imaging was done on EVOS 2 FL (Invitrogen) and analyzed using HCS Cell Studio Analysis Software (ThermoFisher).

### Time-Lapsed Confocal Microscopy

Cells were seeded in Nunc or Cellvis 96 well #1.5 glass bottom plates. Macrophages were stained with CellMask™ Orange (#C10045), and *S. aureus* Newman-CFP bacteria were stained with Sytox™ Green (S7020) for bacterial killing assays. Leica (TCS SP5 spectral confocal microscope) confocal microscope with a thermoregulated chamber at 37C was used for imaging, and ImageJ was used for analysis.

### Cellularity by FACS

Murine lungs and bronchoalveolar lavage (BAL) were collected on day eight (8) of infection trials. BAL was collected using 2mL PBS *via* trachea. Lungs were minced with surgical scissors, then digested in Hank’s Balanced Salt Solution (HBSS) containing liberase (8ug/mL) and DNase I (40ug/mL) for 30-minute incubation. 0.5M EDTA was used to inactivate enzymes. Digested lungs were strained through 40uM cell strainer, then treated with ACK Lysis Buffer for 1 min. Cells were counted and stained with the following panels: A- CD11b FITC, Lys6C PE, Ly6G APC, Fixable Viability Dye eFluor780, B- CD11b FITC, F4/80 PE, CD206 Alexa Fluor 647, CD80 PerCP-Cy 5.5, Fixable Viability Dye eFluor780, C- CD11b FITC, CD11c PE, I-A APC, Fixable Viability Dye eFluor780, D- CD49b FITC, CD19 PE, CD3 APC, Fixable Viability Dye eFluor780.

### Macrophage Polarization

BMDM were stimulated with the following conditions to induce respective M1 and M2 polarizations: M1-IFNg (Pepro Tech, #315-05) and LPS, M2a- IL4 (Pepro Tech, #214-14)/IL13 (Pepro Tech, #210-13), M2b- Immune complex (Polysciences, #23744-5) and LPS, and M2c- IL10 (Pepro Tech, #210-10)/Tgfb (Pepro Tech, #100-21). Cells were induced for 48 hours, then washed and stained. Macrophages were stained with the following: CD86 FITC (Fisher Scientific, BDB553691), Anti-IA PE (Fisher Scientific, 501129471), Anti-CD80 PerCPCy5.5 (Fisher Scientific, BDB560526), Anti-CD206 APC (Fisher Scientific, BDB565250), and fixable viability dye eFluor 780 (eBioscience). Flow Analysis was done on BD LSR II (Franklin Lakes, NJ) and FlowJo (Ashland, OR).

### Lipidomic Profiling by Liquid Chromatography Mass Spectrometry

Lipid mediators were examined by LCMS essentially as described previously (Quehenberger et al., 2011; Tam et al., 2013; Tam et al., 2020). Before lipid metabolite isolation by solid phase extraction (SPE), deuterated standards (Cayman Chemical) were added to 0.9 mL of BAL Methanol was evaporates and the samples reconstituted in a minimal volume of water/acetonitrile (60/40) containing 0.02% v/v acetic acid. Eicosanoids were separated using a Waters Acquity UPLC BEH 1.7 μm 2.1 × 50 mm column using a 4 minute gradient of 99.9% A/B to 75/25 A/B followed by washing and reconditioning. Solvent A is 50/50 water/acetonitrile containing 0.02% acetic acid and solvent B is 50/50 acetonitrile/isopropanol. Eicosanoids were analyzed by a Waters Synapt G2Si QTOF operated in negative-ionization mode *via* MS^e^. Data analysis was performed using UNIFI 1.6 (Waters), MS-DIAL4 (Tsugawa et al., 2020), and Mzmine 2.53 (Pluskal et al., 2010).

## Data Availability Statement

The data presented in the study are deposited in the MetaboLights repository, accession number MTBLS2927.

## Ethics Statement

The animal study was reviewed and approved by IACUC Temple University.

## Author Contributions

VT, RL, BB, and JB contributed to conception and design of the study. RL, JT, and NG-E performed the experiments. BB provided expertise on confocal microscopy. JB performed mass spectrometry analysis. RL and VT wrote the first draft of the manuscript. JB and BB wrote sections of the manuscript. All authors contributed to manuscript revision, read, and approved the submitted version.

## Funding

This work was supported by National Institute of Allergy and Infectious Disease R21 AI142278 and R01 AI168550 (VT).

## Conflict of Interest

The authors declare that the research was conducted in the absence of any commercial or financial relationships that could be construed as a potential conflict of interest.

## Publisher’s Note

All claims expressed in this article are solely those of the authors and do not necessarily represent those of their affiliated organizations, or those of the publisher, the editors and the reviewers. Any product that may be evaluated in this article, or claim that may be made by its manufacturer, is not guaranteed or endorsed by the publisher.

## Supplementary Material

Supplementary Figure 1 Modulating immune response during superinfection. **(A)** Heatmap depicts the hierarchical clustering of transcripts detected (by Nanostring) in macrophages during unstimulated, DHET (14,15-DHET), poly:IC, or poly:IC and DHET stimulation. **(B)** Cluster analysis of co-regulated genes during stimulations described in **(A)**. Red lines represent the mean transcript levels of the clusters. **(C)** Motif enrichment analysis showing promoter sites that have been repressed during superinfection.

Supplementary Figure 2 Cytotoxicity and Luminescence Activity of NFκB decreases during PPARα activation. **(A)** Hoxb8 macrophages with a GFP inducible luciferase reporter were stimulated in combinations with poly:IC (TLR3 agonist), WY14643 (PPARα agonist), or 14,15 DHET (metabolite). Cells were lysed after 1,4 hours, treated with luciferase reagent, and read for 10 seconds per well. n=3 per group and are representative of 3 experiments. **(B)** Hoxb8 macrophages with a GFP inducible luciferase reporter were stimulated in combinations with LPS (TLR4 agonist), WY14643 (PPARα agonist), or 14,15 DHET (metabolite). Cells were lysed after 6 hours, treated with luciferase reagent, and read for 10 seconds per well. n=4-6 per group. **(C)** Hoxb8 macrophages were stimulated with varying concentrations of WY14643. Cells were stained with AnnexinV and Propidium iodide to determine early (AnnexinV+ PI-) and late (AnnexinV+ PI+) apoptosis by FACS. Two way ANOVA with multiple comparisons were performed to determine statistical significance (*P ≤ 0.05; **P ≤ 0.01; ***P ≤ 0.001; ****P ≤ 0.0001).

Supplementary Figure 3 Immunoblots of NFκB and PPARα. **(A)** Immunoblotting of Hoxb8 macrophages (C57BL/6 and *Ppara* ^–/–^) stimulated with mock (DMSO), LPS, LPS and WY14643 against NFκB P65, phospho-NFκB P65 (S536) and β-actin. **(B)** Immunoblotting of Hoxb8 macrophages (C57BL/6 and *Ppara* ^–/–^) against PPARα. Arrow indicates PPARα (~52kDa).

Supplementary Figure 4 Nuclear Translocation of NFκB and PPARα. Representative images from digital microscopy of nuclear translocation of NFκB and PPARα are shown. NFκB is shown in red (CY5), PPARα is shown in green (AlexaFluor 488), and the nucleus is stained blue (DAPI).

Supplementary Figure 5 Nuclear Translocation of NFκB and PPAR. (single channel images). Representative images from digital microscopy of nuclear translocation of NFκB and PPARα are shown. Single channel images of DAPI (nucleus), PPARα, and NFκB.

Supplementary Figure 6 Flow cytometry gating strategy for quantifying cells from bronchoalveolar lavage samples. After debris was gated out (FSC-A v SSC-A), live cells (Fixable viability dye-) and singlets (FSC-H v FSC-A) were selected. Neutrophils (CD11b+ Ly6G+), Inflammatory monocytes (CD11b+, Ly6C+ Ly6G-), CD11b+ F4/80+ macrophages and CD206+ M2 cells, CD11c+MHCII+ DC, CD19+ B cells, CD3+ T cells, and CD49b+ NK cells were quantified.

Supplementary Figure 7 Cellularity of superinfection. Bar graphs depict the percentages of CD11b+ Ly6C+ inflammatory monocytes **(A)**, neutrophils **(B)**, DC **(C)**, T cells **(D)**, NK cells **(E)**, and B-cells **(F)** isolated from the bronchoalveolar lavage from infected lungs during influenza, *S.aureus*, or superinfection. Two way ANOVA with multiple comparisons were performed to determine statistical significance (*P ≤ 0.05; **P ≤ 0.01; ***P ≤ 0.001; ****P ≤ 0.0001).

Supplementary Figure 8 Transcriptional responses of macrophage polarization change during PPARα activation. Bar graphs depict transcript levels (mean+/- SEM) as measured by RT-PCR from C57BL/6 macrophages stimulated with IFNγ LPS (M1), IL4/IL13 (M2a), and immune complex and LPS (M2b) with (red) or without (black) WY14643. Two way ANOVA with multiple comparisons were performed to determine statistical significance (*P ≤ 0.05; **P ≤ 0.01; ***P ≤ 0.001; ****P ≤ 0.0001).

Supplementary Figure 9 Increased expression of CD206 upon WY14643 stimulation during M2b polarization. Bar graph depicts the mean fluorescent intensity of CD206 (mean +/- SEM) of macrophages stimulated with mock (media), WY14643, IL4/IL13 (M2a), Immune complex [IC] + LPS (M2b). Two way ANOVA with multiple comparisons were performed to determine statistical significance (*P ≤ 0.05; **P ≤ 0.01; ***P ≤ 0.001; ****P ≤ 0.0001). n=3 per group and are representative of 3 experiments.
