# Supplementary figures and images for: Eicosanoid-Activated PPARα Inhibits NFκB-Dependent Bacterial Clearance During Post-Influenza Superinfection
Source: Front Cell Infect Microbiol. 2022 Jul 4;12:881462. doi: 10.3389/fcimb.2022.881462 (PMC9289478; doi:10.3389/fcimb.2022.881462)

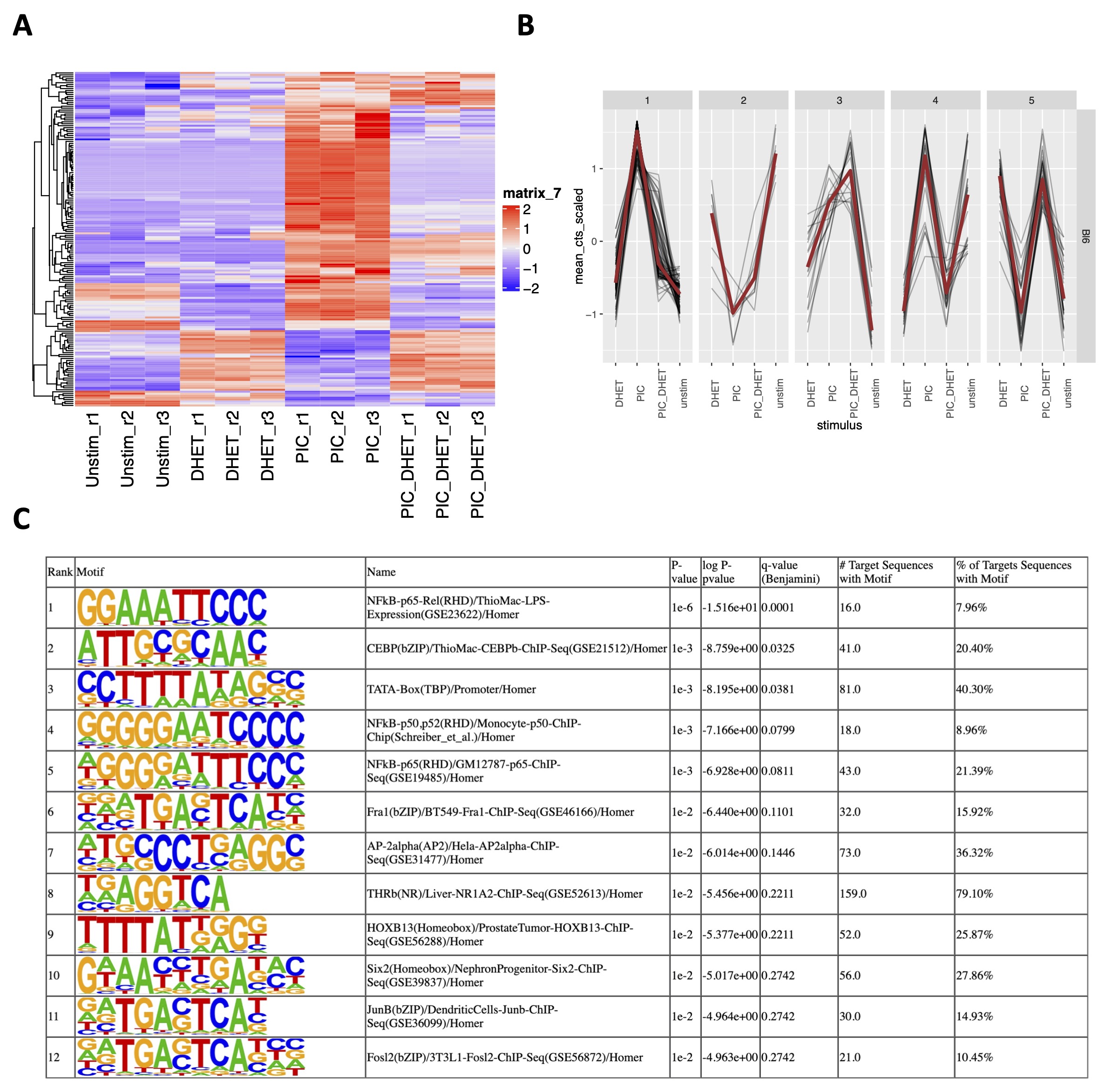

Supplement: Supplementary Figure 1 — Modulating immune response during superinfection. (A) Heatmap depicts the hierarchical clustering of transcripts detected (by Nanostring) in macrophages during unstimulated, DHET (14,15-DHET), poly:IC, or poly:IC and DHET stimulation. (B) Cluster analysis of co-regulated genes during stimulations described in (A). Red lines represent the mean transcript levels of the clusters. (C) Motif enrichment analysis showing promoter sites that have been repressed during superinfection. [file Image_1.jpg]

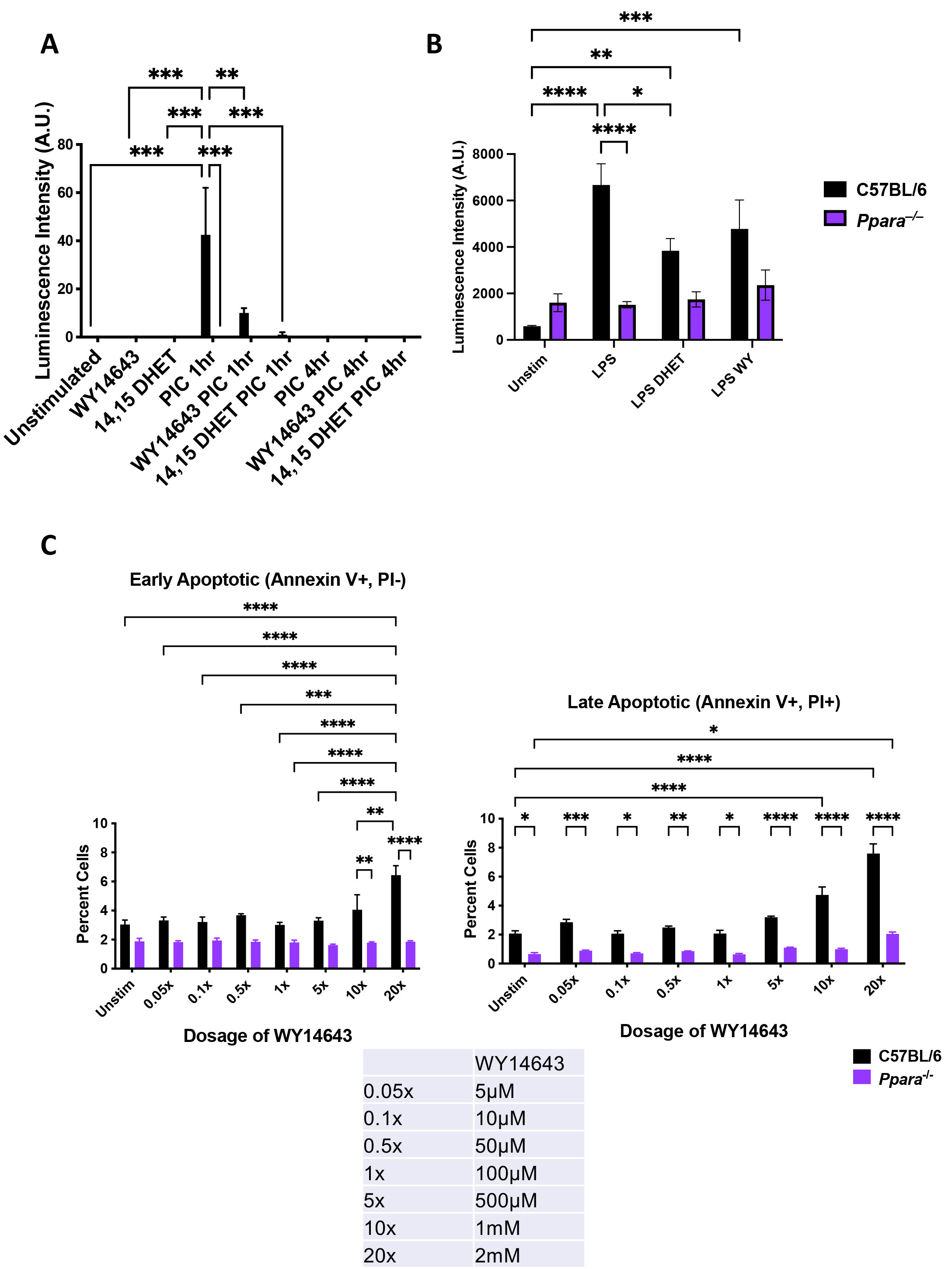

Supplement: Supplementary Figure 2 — Cytotoxicity and Luminescence Activity of NFκB decreases during PPARα activation. (A) Hoxb8 macrophages with a GFP inducible luciferase reporter were stimulated in combinations with poly:IC (TLR3 agonist), WY14643 (PPARα agonist), or 14,15 DHET (metabolite). Cells were lysed after 1,4 hours, treated with luciferase reagent, and read for 10 seconds per well. n=3 per group and are representative of 3 experiments. (B) Hoxb8 macrophages with a GFP inducible luciferase reporter were stimulated in combinations with LPS (TLR4 agonist), WY14643 (PPARα agonist), or 14,15 DHET (metabolite). Cells were lysed after 6 hours, treated with luciferase reagent, and read for 10 seconds per well. n=4-6 per group. (C) Hoxb8 macrophages were stimulated with varying concentrations of WY14643. Cells were stained with AnnexinV and Propidium iodide to determine early (AnnexinV+ PI-) and late (AnnexinV+ PI+) apoptosis by FACS. Two way ANOVA with multiple comparisons were performed to determine statistical significance (*P ≤ 0.05; **P ≤ 0.01; ***P ≤ 0.001; ****P ≤ 0.0001). [file Image_2.jpeg]

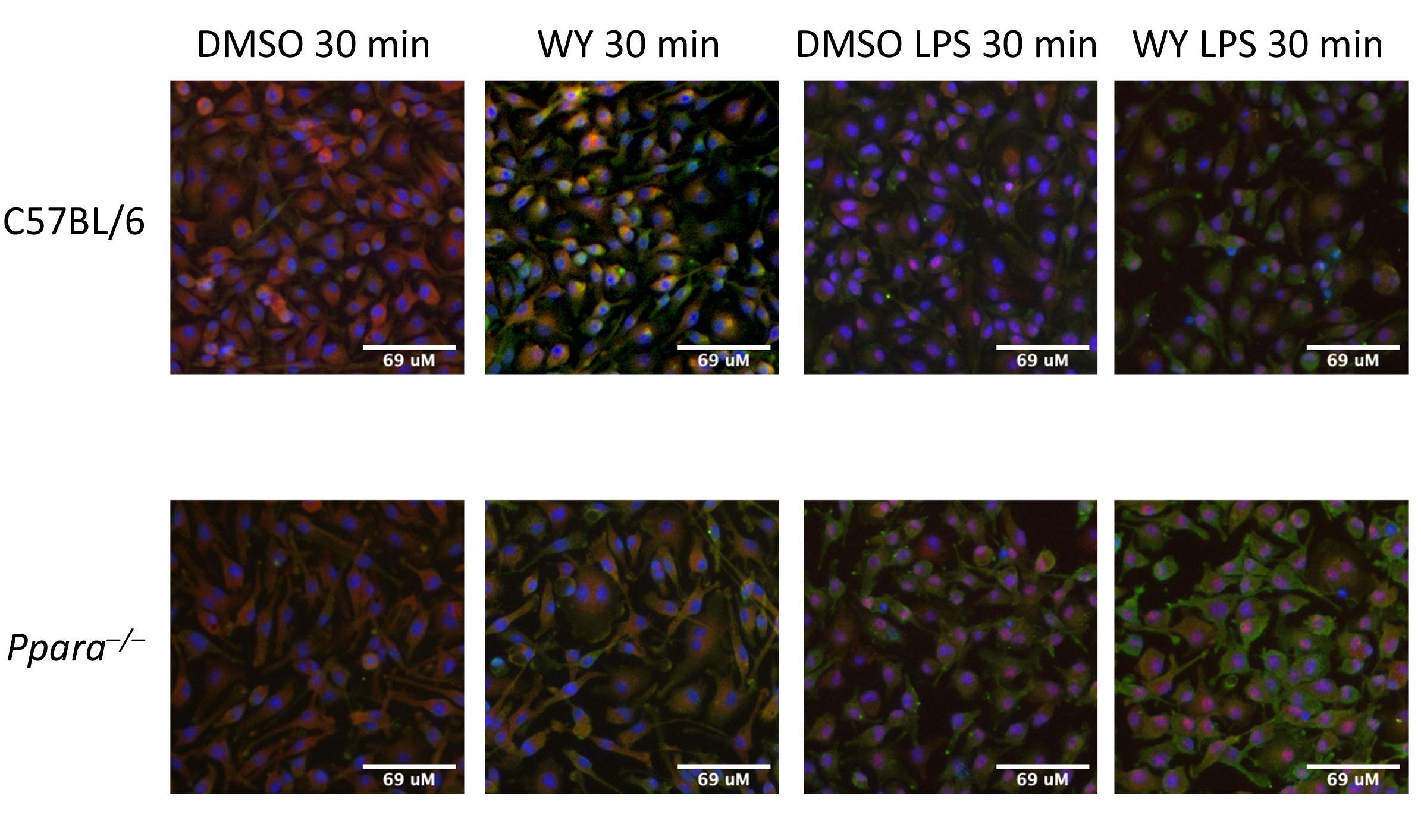

Supplement: Supplementary Figure 4 — Nuclear Translocation of NFκB and PPARα. Representative images from digital microscopy of nuclear translocation of NFκB and PPARα are shown. NFκB is shown in red (CY5), PPARα is shown in green (AlexaFluor 488), and the nucleus is stained blue (DAPI). [file Image_4.jpeg]

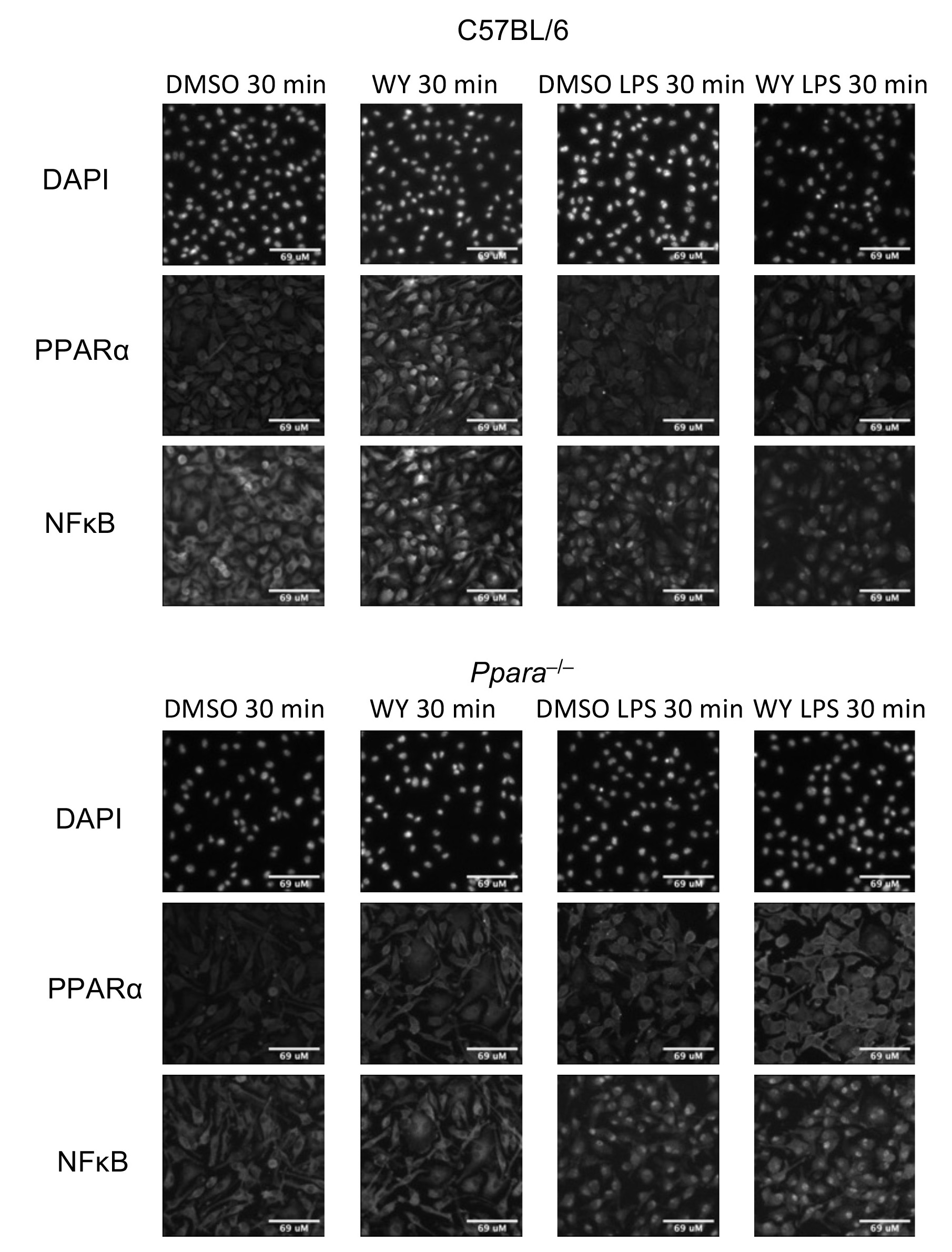

Supplement: Supplementary Figure 5 — Nuclear Translocation of NFκB and PPAR. (single channel images). Representative images from digital microscopy of nuclear translocation of NFκB and PPARα are shown. Single channel images of DAPI (nucleus), PPARα, and NFκB. [file Image_5.jpeg]

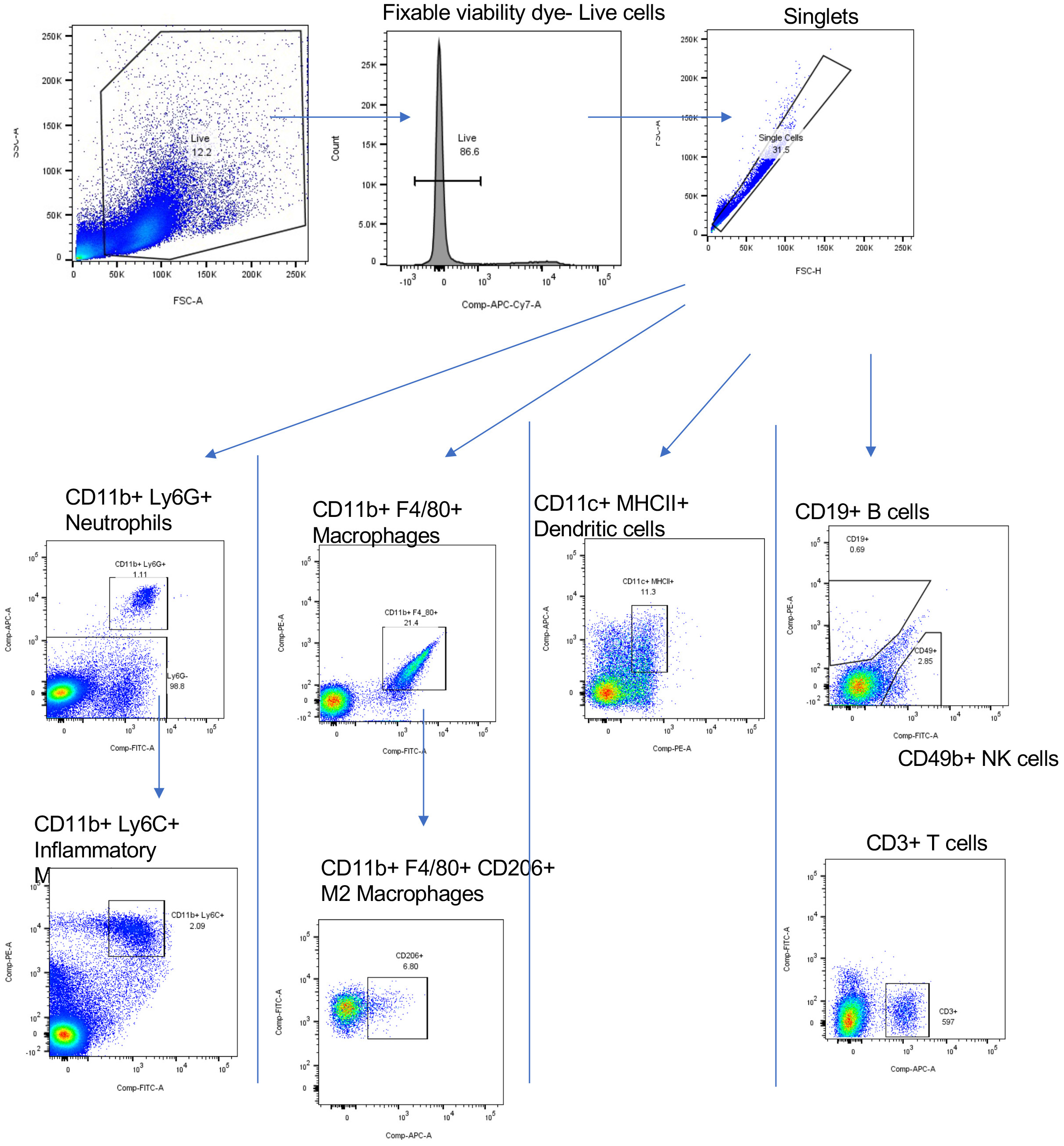

Supplement: Supplementary Figure 6 — Flow cytometry gating strategy for quantifying cells from bronchoalveolar lavage samples. After debris was gated out (FSC-A v SSC-A), live cells (Fixable viability dye-) and singlets (FSC-H v FSC-A) were selected. Neutrophils (CD11b+ Ly6G+), Inflammatory monocytes (CD11b+, Ly6C+ Ly6G-), CD11b+ F4/80+ macrophages and CD206+ M2 cells, CD11c+MHCII+ DC, CD19+ B cells, CD3+ T cells, and CD49b+ NK cells were quantified. [file Image_6.jpeg]

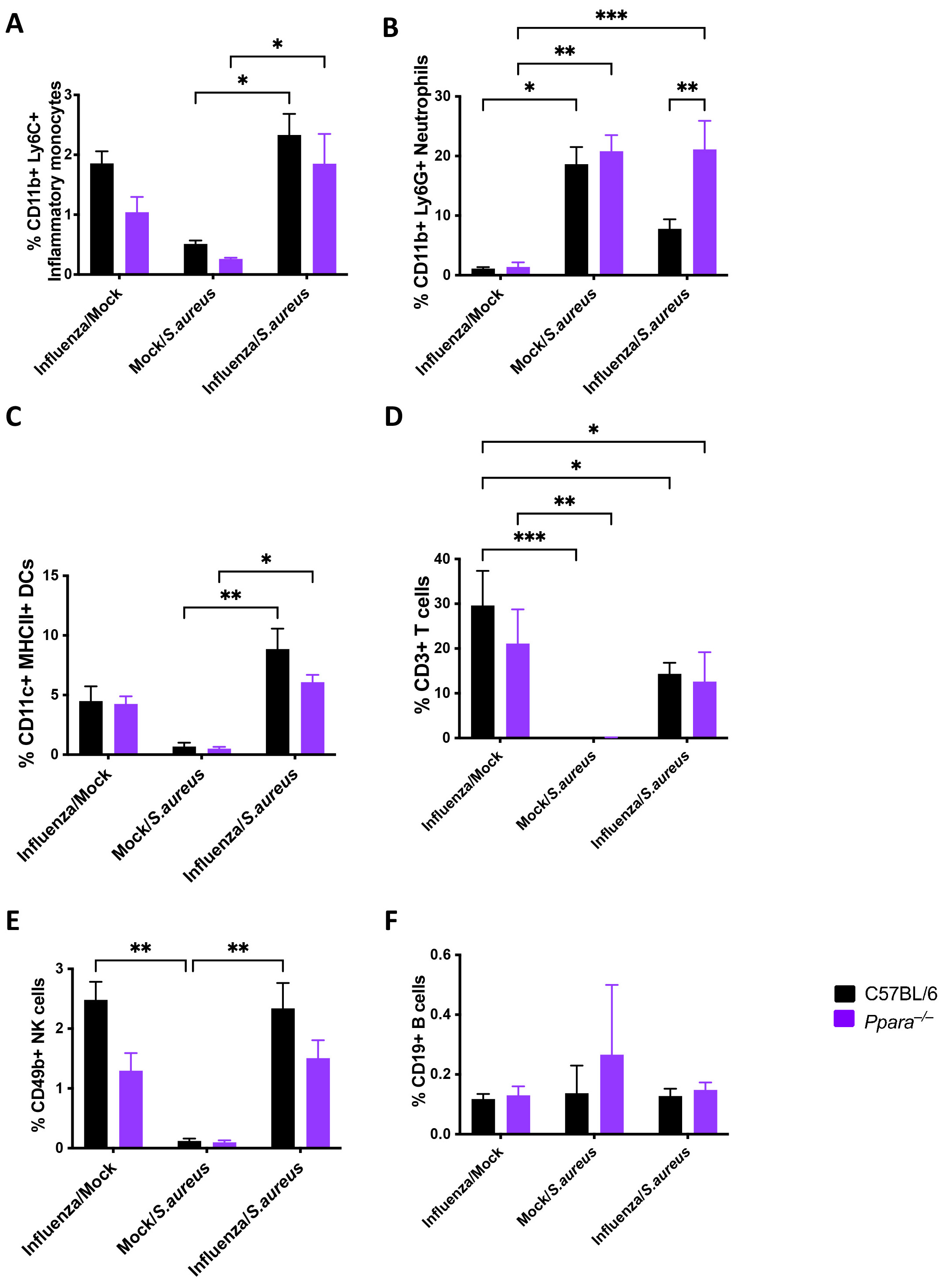

Supplement: Supplementary Figure 7 — Cellularity of superinfection. Bar graphs depict the percentages of CD11b+ Ly6C+ inflammatory monocytes (A), neutrophils (B), DC (C), T cells (D), NK cells (E), and B-cells (F) isolated from the bronchoalveolar lavage from infected lungs during influenza, S.aureus, or superinfection. Two way ANOVA with multiple comparisons were performed to determine statistical significance (*P ≤ 0.05; **P ≤ 0.01; ***P ≤ 0.001; ****P ≤ 0.0001). [file Image_7.jpeg]

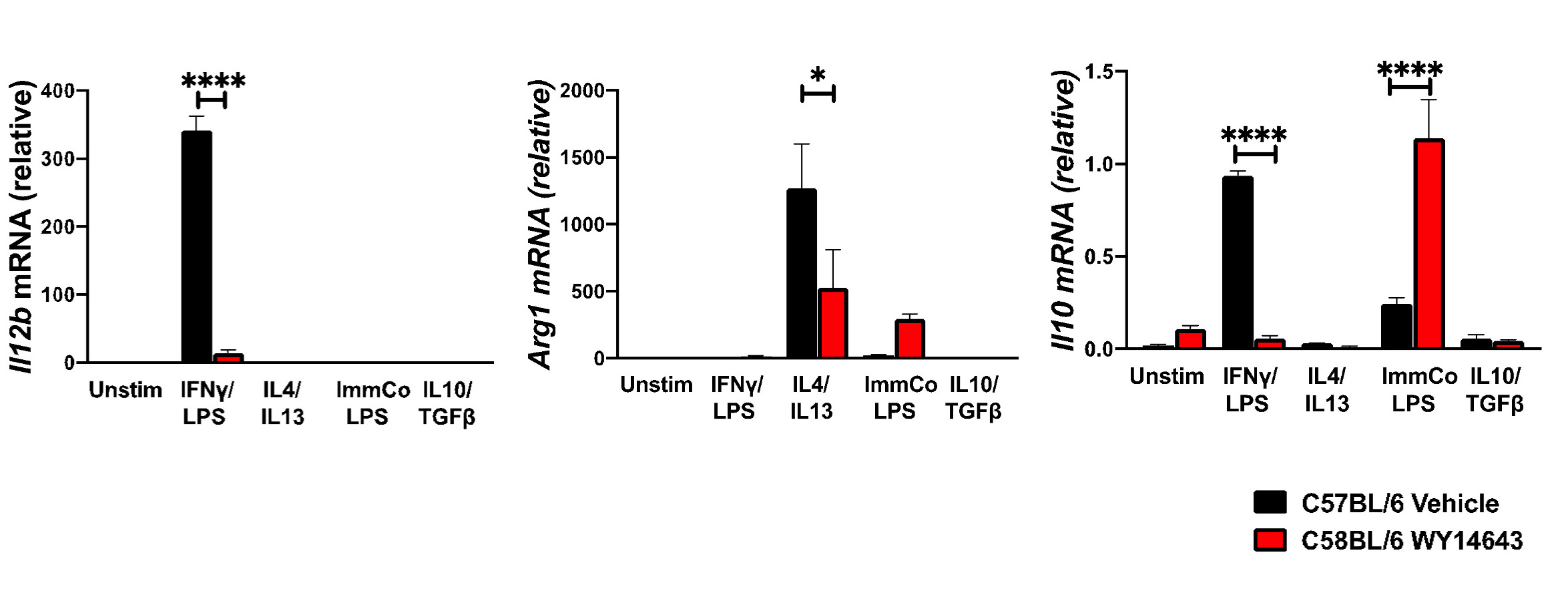

Supplement: Supplementary Figure 8 — Transcriptional responses of macrophage polarization change during PPARα activation. Bar graphs depict transcript levels (mean+/- SEM) as measured by RT-PCR from C57BL/6 macrophages stimulated with IFNγ LPS (M1), IL4/IL13 (M2a), and immune complex and LPS (M2b) with (red) or without (black) WY14643. Two way ANOVA with multiple comparisons were performed to determine statistical significance (*P ≤ 0.05; **P ≤ 0.01; ***P ≤ 0.001; ****P ≤ 0.0001). [file Image_8.jpeg]

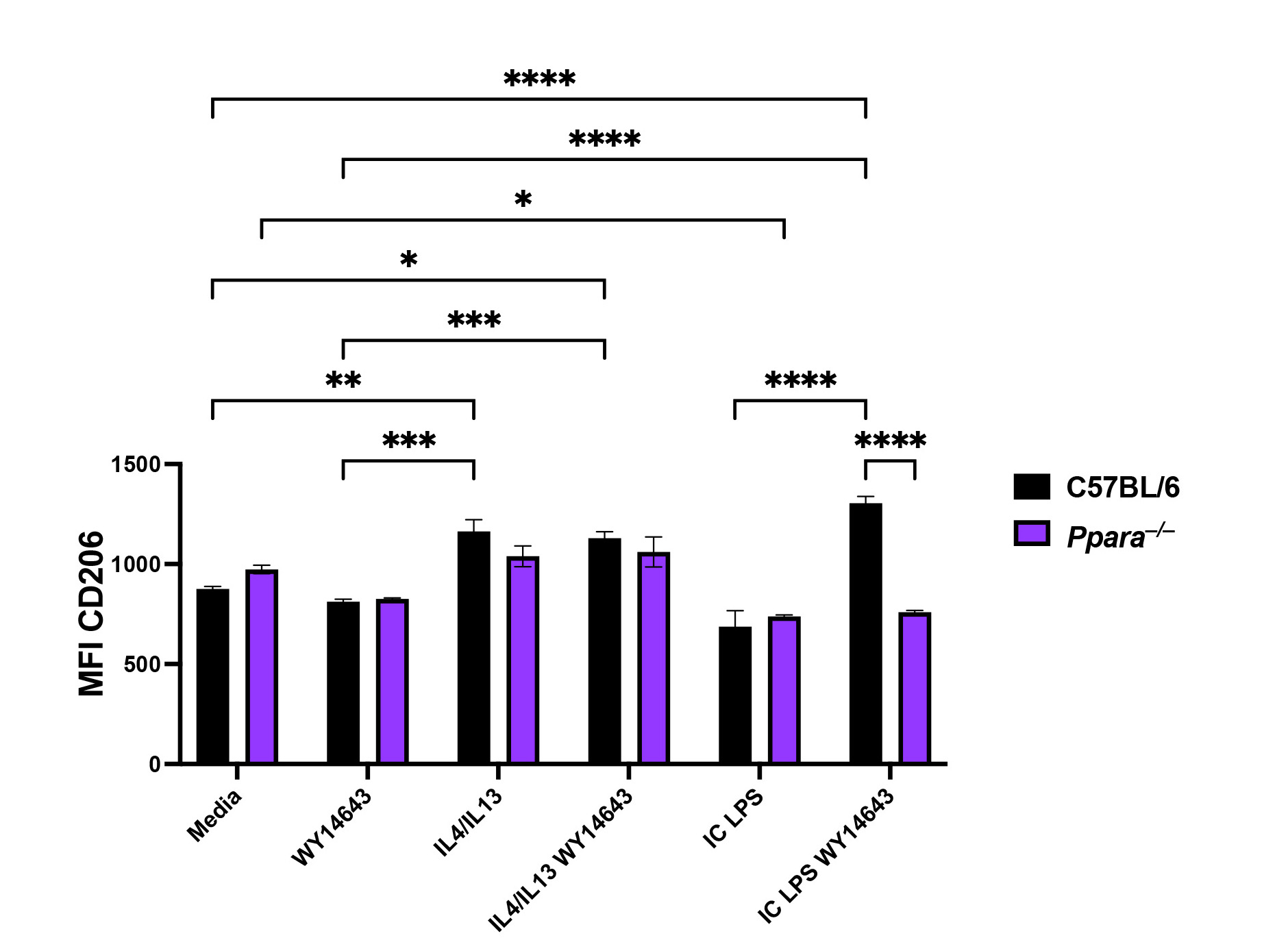

Supplement: Supplementary Figure 9 — Increased expression of CD206 upon WY14643 stimulation during M2b polarization. Bar graph depicts the mean fluorescent intensity of CD206 (mean +/- SEM) of macrophages stimulated with mock (media), WY14643, IL4/IL13 (M2a), Immune complex [IC] + LPS (M2b). Two way ANOVA with multiple comparisons were performed to determine statistical significance (*P ≤ 0.05; **P ≤ 0.01; ***P ≤ 0.001; ****P ≤ 0.0001). n=3 per group and are representative of 3 experiments. [file Image_9.jpeg]
