# Supplementary material for: Eicosanoid-Activated PPARα Inhibits NFκB-Dependent Bacterial Clearance During Post-Influenza Superinfection
Source: Front Cell Infect Microbiol. 2022 Jul 4;12:881462. doi: 10.3389/fcimb.2022.881462 (PMC9289478; doi:10.3389/fcimb.2022.881462)
Supplement: Supplementary file 10 [file Table_1.docx]

| **REAGENT or RESOURCE** | **SOURCE** | **IDENTIFIER** |
| --- | --- | --- |
| Antibodies | | |
| Anti-NFκB p65 Antibody (F-6) | Santa Cruz Biotechnology | Cat#: SC-8008  RRID:AB_628017 |
| PPAR alpha/NR1C1 [p Ser12] Antibody | Novus Biologicals | Cat#: NB120-3484  RRID:AB_791531 |
| CD11b Rat anti-Mouse, FITC, Clone: M1/70 | BD Biosciences | Cat#: BDB557396  RRID: AB_396679 |
| F4/80 Rat anti-Mouse, PE, Clone: BM8 | eBioscience | Cat#:50-107-86  RRID:AB_465922 |
| CD206 Rat anti-Mouse, Alexa Fluor 647, Clone: MR5D3 | BD Biosciences | Cat#: BDB565250  RRID: AB_2739133 |
| Ly-6G Rat anti-Mouse, APC, Clone: 1A8 | BD Biosciences | Cat#: BDB560599  RRID: AB_1727560 |
| Ly-6C Rat anti-Mouse, PE, Clone: AL-21 | BD Biosciences | Cat#: BDB560592  RRID: AB_1727556 |
| CD11c Hamster anti-Mouse, PE, Clone: HL3 | BD Biosciences | Cat#: BD553802  RRID: AB_395061 |
| MHC Class II (I-A/I-E) Rat anti-mouse, APC Clone: M5/M114.15.2 | eBioscience | Cat#: 50-112-9471  RRID: AB_469454 |
| CD80 (B7-1) Hamster anti-Mouse, PerCP-Cy5.5, Clone: 16-10A1 | BD Biosciences | Cat#: BDB560526  RRID:AB_1727514 |
| FITC Rat anti-Mouse CD49b Clone: DX5 | BD Biosciences | Cat#: BD553857  RRID: AB_395093 |
| CD3e Hamster anti-Mouse, APC, Clone: 145-2C11 | BD Biosciences | Cat#: BDB553066  RRID: AB_398529 |
| CD19 Rat anti-Mouse, PE, Clone: 1D3 | BD Biosciences | Cat#: BDB553786  RRID: AB_395050 |
| I-A/I-E Rat anti-Mouse, PE, Clone: M5/114.15.2 | BD Biosciences | Cat#: 557000  RRID:AB_396546 |
| Goat anti-Mouse IgG (H+L) Cross-Absorbed Secondary Antibody, Cyanine5 | Invitrogen | Cat#: A10524  RRID: AB_2534033 |
| Goat anti-Rabbit Secondary Antibody, Alexa Fluor 488 | Invitrogen | Cat#: A11054  RRID: AB_2576217 |
| PPAR alpha monoclonal antibody (3B6/PPAR) | Invitrogen | Cat#: MA1-822  RRID:AB_2165745 |
| Bacterial and virus strains | | |
| Staphylococcus aureus, Strain: Newman | Dr. Ferric Fang (University of Washington) |  |
| Staphylococcus aureus, Strain: RN4220 | Bei RESOURCES | NR-45946 |
| Influenza PR8 | Dr. Paul Thomas (St. Jude Children’s Hospital) |  |
| Staphylococcus aureus, Strain: Newman pTH2 (sarA P1::CFP::pCM29) | This study | This study |
| Staphylococcus aureus, Strain: Newman pRN11 (sarA P1::mCherry::pCM29) | This study | This study |
| Chemicals, peptides, and recombinant proteins | | |
| WY14643 | Sigma Aldrich | Cat#: C7081 |
| Fixable Viability Dye 780 | eBioscience | Cat#: 501129035 |
| Recombinant Murine IFNγ | Pepro Tech | Cat#: 315-05 |
| LPS from *Salmonella minnesota* R595 (Re) | VWR International | Cat#: 102946-568 |
| Recombinant Murine IL4 | Pepro Tech | Cat#: 214-14 |
| Recombinant Murine IL13 | Pepro Tech | Cat#: 210-13 |
| Ova |  |  |
| Anti-Ovalbumin (egg white) in rabbit, IgG fraction | Polysciences | Cat#:23744-5 |
| Critical commercial assays | | |
| Direct-zol 96 RNA Preps | Zymo Research | Cat#: R2061 |
| Experimental models: Cell lines | | |
| L-929 Cells | ATCC | NCTC clone 929 |
| Experimental models: Organisms/strains | | |
| C57BL/6 mouse | The Jackson Laboratory | Stock#: 000664 |
| B6;129S4-*Ppara^tm1Gonz^*/J mouse | The Jackson Laboratory | Stock#: 008154 |
| Recombinant DNA | | |
| Plasmid: pTH2 (sarA P1::CFP::pCM29) | Addgene | 84452 |
| Plasmid: pRN11 (sarA P1::mCherry::pCM29) | Addgene | 84455 |
| Plasmid: pHAGE NFκB-TA-LUC-UBC-GFP-W | Addgene | 49343 |
| Software and algorithms | | |
| FlowJo 10.7.1 | Becton Dickinson & Company | <https://www.flowjo.com/solutions/flowjo> |
| BD FACSDiva^TM^ Software | BD Biosciences- US | <https://www.bdbiosciences.com/en-us/instruments/research-instruments/research-software/flow-cytometry-acquisition/facsdiva-software> |
| Evos FL Auto 2 Cell Imaging System | ThermoFisher Scientific | <https://www.thermofisher.com/us/en/home/technical-resources/software-downloads/evos-fl-auto2-imaging-system-software-download.html> |
| High Content Screening-Analysis (HCS) | ThermoFisher Scientific | <https://www.thermofisher.com/us/en/home/life-science/cell-analysis/cellular-imaging/high-content-screening.html> |
| GraphPad Prism 9.1.1 | GraphPad Software | <https://www.graphpad.com/scientific-software/prism/> |
| ImageJ | National Institutes of Health | <https://imagej.nih.gov/ij/index.html> |
| Image Studio Lite | LI-COR Biosciences | <https://www.licor.com/bio/image-studio-lite/download> |
| StepOne^TM^ Software | ThermoFisher Scientific | <https://www.thermofisher.com/us/en/home/technical-resources/software-downloads/StepOne-and-StepOnePlus-Real-Time-PCR-System.html> |
| Leica Application Suite- Advanced Fluorescence 2.6.0.7266 | Leica Microsystems | <https://www.leica-microsystems.com/products/microscope-software/> |
| Waters Unifi 1.8 | Waters | <https://www.waters.com/waters/en_US/UNIFI-Scientific-Information-System/nav.htm?cid=134801359&locale=en_US> |
| MZmine 2.53 | MZmine | <http://mzmine.github.io/> |
| MS-DIAL4 | RIKEN CSRS | <http://prime.psc.riken.jp/compms/msdial/main.html> |
| R 3.6.2 | R project | <https://www.r-project.org/> |
| R studio 1.3.959 | R studio | <https://www.rstudio.com/> |
| ComplexHeatmap (R package) |  | <https://jokergoo.github.io/ComplexHeatmap-reference/book/> |
| BioRender | BioRender | <https://biorender.com> |
